# In Situ Hybridization (RNAscope) Detection of Bluetongue Virus Serotypes 10 and 17 in Experimentally Co-Infected *Culicoides sonorensis*

**DOI:** 10.3390/pathogens12101207

**Published:** 2023-09-30

**Authors:** Molly Carpenter, AnaMario Benavides Obon, Jennifer Kopanke, Justin Lee, Kirsten Reed, Tyler Sherman, Case Rodgers, Mark Stenglein, Emily McDermott, Christie Mayo

**Affiliations:** 1 Department of Microbiology, Immunology, and Pathology, Colorado State University, 1601 Campus Delivery, Fort Collins, CO 80526, USA; molly.carpenter@colostate.edu (M.C.); kirsten.reed@colostate.edu (K.R.); case1prod@gmail.com (C.R.); mark.stenglein@colostate.edu (M.S.); 2 Diagnostic Medicine Center, Colorado State University, 2450 Gillette Drive, Fort Collins, CO 80526, USA; ana.benavides_obon@colostate.edu (A.B.O.); tyler.sherman@colostate.edu (T.S.); 3 Department of Comparative Medicine, Oregon Health & Science University, Portland, OR 97239, USA; kopanke@ohsu.edu; 4 Genomic Sequencing Laboratory, Centers for Disease Control and Prevention, Atlanta, GA 30333, USA; psd8@cdc.gov; 5 Department of Entomology and Plant Pathology, University of Arkansas, Fayetteville, AR 72701, USA; emcdermo@uark.edu

**Keywords:** bluetongue virus, co-infection, *Culicoides*, in situ hybridization

## Abstract

Bluetongue virus (BTV) is a segmented, double-stranded RNA virus transmitted by *Culicoides* biting midges. Infection of domestic and wild ruminants with BTV can result in a devastating disease and significant economic losses. As a virus with a segmented genome, reassortment among the BTV serotypes that have co-infected a host may increase genetic diversity, which can alter BTV transmission dynamics and generate epizootic events. The objective of this study was to determine the extent of dissemination and characterize the tropism of BTV serotypes 10 and 17 in co-infected *Culicoides sonorensis*. Midges were exposed to both BTV serotypes via blood meal and processed for histologic slides 10 days after infection. An in situ hybridization approach was employed using the RNAscope platform to detect the nucleic acid segment 2 of both serotypes. Observations of the mosaic patterns in which serotypes did not often overlap suggest that co-infection at the cellular level may not be abundant with these two serotypes in *C. sonorensis*. This could be a consequence of superinfection exclusion. Understanding BTV co-infection and its biological consequences will add an important dimension to the modeling of viral evolution and emergence.

## 1. Introduction

Bluetongue virus (BTV) is an arbovirus, listed by the World Organisation for Animal Health, that can infect both domestic and wild ruminants, with sheep being particularly susceptible. As an animal disease of international concern, there can be severe economic consequences for livestock stakeholders through production and trade losses [1,2]. The arthropod responsible for transmitting BTV is the *Culicoides* biting midge (Diptera: Ceratopogonidae), with the geographic range of BTV defined by the presence of competent *Culicoides* species [3,4]. Concerningly, there were recent incursions of novel serotypes of BTV into historically enzootic regions including the southeastern United States (US), South America, Israel, and Australia [5,6,7,8,9]. Explanations for these observed expansions include increased vector distribution, secondary to climate variability, and virus evolution, resulting in altered transmission dynamics [10,11,12].

The evolution of BTV is of particular interest, as it is a segmented double-stranded RNA virus that is taxonomically organized into the order *Reovirales*, the family *Sedaroviridae,* and the genus *Orbivirus* [13,14,15]. The serotype is defined by the viral protein 2 outer capsid structure (VP2) that is encoded by nucleic acid segment 2, which elicits the antibody response in the livestock host [16,17]. There are currently >29 recognized BTV serotypes [18,19,20]. As BTV has a segmented genome, if a cell is infected with two different parental virus strains, evolution via reassortment is possible [21,22]. The reassortment of BTV was abundantly characterized in field surveillance sequencing [23,24]. However, BTV dissemination, tissue tropism, and the extent of cellular co-infection occurrence in the midge host are largely unknown. The application of in situ hybridization (ISH) technology could advance knowledge of the virus presence within the midge host’s tissues. ISH technology amplifies the signal of specific nucleic acid sequences, permitting the visualization of pathogen nucleic acid in a histological section [25,26].

In this study, we applied RNAscope technology (a commercial ISH platform by Advanced Cell Diagnostics; ACD) to *Culicoides sonorensis*, exposed by blood meal to two different BTV serotypes (BTV serotype 10 (BTV-10) and BTV serotype 17 (BTV-17)), to characterize virus tissue tropism and the potential for cellular co-infection. RNAscope probes designed for segment 2 of BTV-10 and BTV-17 were applied to histological cross sections of BTV-exposed *C. sonornesis*. Overall, there was success in detecting the presence of BTV-10 and BTV-17 via this technique. Informative patterns in BTV tissue tropisms and localizations were observed, including a presence in the salivary glands and ommatidia.

## 2. Materials and Methods

### 2.1. Culicoides Sonorensis and Viruses

The *C. sonorensis* used in this study were from the AK colony provided by the USDA ARS, Manhattan, KS, USA. This colony is derived from wild midges collected in Idaho in 1973 [27]. Upon arrival, *C. sonorensis* were acclimated for at least 24 h at 25 ± 2 °C on a 12:12 h light cycle and fed ad libitum 10% sucrose solution [27,28].

BTV-10 (bluetongue virus, type 10, strain 8, ATCC VR-187) and BTV-17 (BTV serotype 17 CO 2018) viral strains were used for the infections of *C. sonorensis* due to their endemicity to the United States and their demonstrable ability to infect AK colony *C. sonorensis* [unpublished data]. BTV-10 was acquired from an ATCC strain isolated from a sheep in California in 1952 and passaged eight times on BHK 21 cells [29]. The BTV-17 strain was isolated from the whole blood of a sheep in Colorado in 2018 on CuVaW3 cells and, subsequently, passaged two times on BHK 21 cells [30]. Infectious titers for BTV-10 and BTV-17 were determined by the 50% tissue culture infectious dose (TCID_50_/mL) assay, as described by Kopanke et al. using the Reed–Muench equation [31,32].

### 2.2. C. sonorensis Infection and Maintenance

Mechanically defibrinated sheep blood (Hemostat Laboratories, Dixon, CA, USA, or Lampire Biological Laboratories, Everett, PA, USA) was screened for the presence of BTV via qRT-PCR and for BTV antibodies via BTV cELISA (VMRD, Pullman, WA, USA), as previously described by Kopanke et al. [31]. Single BTV-10 or BTV-17 infections and BTV-10 and BTV-17 co-infections were established via virus-spiked blood meals [31]. For each single infection and co-infection treatment group, BTV stock was diluted in defibrinated sheep blood to produce a final titer of 1 × 10^5^ TCID_50_/mL (Supplementary Table S1). For the negative control group, defibrinated sheep blood was combined with inoculum media of the same volume as that used in the infectious blood-feeding treatment groups. Nulliparous *C. sonorensis* were infected via BTV-spiked blood meal 3–4 days post-emergence. Glass bell feeders equipped with parafilm membranes were used to supply 37 °C-prepared blood treatments. Female midges were given access to prepared blood for 80 min in the presence of male midges. After blood feeding, *C. sonorensis* were immobilized by being placed into a −20 °C freezer for 5 min. Engorged *C. sonorensis* were then sorted on a modified chill table and retained for this study by grouping approximately 100 *C. sonorensis* per container.

For the remainder of the study, *C. sonorensis* were housed in paper tube containers (Rigid Paper Tube Corporation, Wayne, NJ, USA) with sheer nylons stretched over the lid to provide air exchange and a mechanism for feeding. Sucrose solution (10% *w/v*) was available at all times through a cotton wick placed in each container. *C. sonorensis* were maintained at 25 ± 2 °C with a 12:12 h light cycle.

Ten days post-infection, live *C. sonorensis* were sacrificed for processing via immobilization in a −20 °C freezer, placed in CellSafe capsules inserted in biopsy cassettes, and then immersed in 10% buffered formalin. *C. sonorensis* were embedded in paraffin, serially sectioned, and mounted on SuperFrost Plus Slides (Fisher Scientific, Pittsburgh, PA, USA, Cat. No. 12-550-15) by the Colorado State University Biopsy and Histopathology Service Department, as described by the ACD Formalin-Fixed Paraffin-Embedded (FFPE) Sample Preparation and Pretreatment manual for the RNAscope 2.5 Assay (document number 322452).

### 2.3. In Situ Hybridization: RNAscope 2.5 HD Chromogenic Duplex Detection

Chromogenic visualization of BTV-10 and BTV-17 nucleic acids was accomplished with RNA in situ hybridization (RNA-ISH) technology using the commercially marketed RNAscope 2.5 HD Chromogenic Duplex Detection Kit (Chromogenic) (ACD). Sequences for all 10 segments of BTV-17 and BTV-10 used in this study were provided to ACD for probe design targets and to make sure probes did not cross-react with other BTV segments (Supplementary Table S2). The nucleic acid segment 2 that encodes VP2 of BTV-17 (nucleotides 1801-2839, OQ798199) was the target for the Channel 1 (C1) probes. VP2 of BTV-10 (nucleotides 184–1168; GenBank accession number JQ740772, with the ability to cross-react with MW456748 BTV-10 California 1952) was the target of the Channel 2 (C2) probes. The positive control probes designed for Channel 1 targeted the protein elongation factor 1-beta (*EF1b*, nucleotides 2-954; GenBank accession number GAWM01010754), which demonstrated high expression across different conditions with high cycle threshold (Ct) values in diverse tissue types [33]. The positive control probes designed for Channel 2 targeted the vacuolar ATPase gene (nucleotides 2-668; GenBank accession number AY753855), which was sequenced from the same AK *C. sonorensis* colony and was used as an internal positive extraction control for previous *C. sonorensis* studies [34,35]. Probes targeting the dihydrodipicolinate reductase (*DapB*) gene were provided by ACD as a negative control and to evaluate for non-specific staining, as it was expected to be absent in study samples. A table of probe targets is provided (Supplementary Table S3). All probes were designed and manufactured by ACD. Probes were designed to not cross-react with each other, other segments of BTV strains used in the study, or known *C. sonorensis* sequences.

One slide from each of the serially sectioned *C. sonorensis* treatment groups was stained with hematoxylin and eosin to evaluate and determine optimal cross sections and assist in identifying anatomical structures. Sample preparation, pretreatment, and staining procedures were conducted according to the Formalin-Fixed Paraffin-Embedded (FFPE) Sample Preparation and Pretreatment manual for the RNAscope 2.5 Assay Part 1 (document number 322452) and RNAscope 2.5 HD Duplex Detection Kit (Chromogenic) Part 2 (document number 322500-USM) with probes, reagents, and the HybEZ II Oven recommended by ACD. Two serial cross sections from each *C. sonorensis* were processed from each infection treatment group as technical replicates. There were 15 midges for the BTV-10 infection treatment group, 13 midges for the BTV-17 infection treatment group, and 14 midges for the BTV-10 and BTV-17 co-infection treatment group. 

Slide Pretreatment. Slides were dried for 1 h at 60 °C, deparaffinized in xylene twice for 5 min, washed twice in 100% alcohol for 1 min, and then dried for approximately 5 min. Tissue sections were covered with hydrogen peroxide and incubated for 10 min. Hydrogen peroxide was removed from the slide and dipped in distilled water several times to remove any remaining reagent. The slides were boiled at 100 °C for 15 min in beakers containing the target retrieval reagents. After rinsing with distilled water, slides were immersed in 100% ethanol for 3 min, followed by a drying period of approximately 5 min. RNAscope Protease Plus was applied for 15 min at 40 °C.

Staining. Excess liquid was removed from the slides, and 4 drops of the probe mix (C1: BTV-17 and C2: BTV-10) were applied to treatment group slides (including negative control group slides). Four drops of control probes (negative: DapB; positive control C1: Cs0-TSA-m4178; positive control C2: Cso-V-ATPase16) were applied to separate control slides to affirm the success of the hybridization procedure. After application of probes, slides were incubated for 2 h at 40 °C.

Hybridization. Signal amplification scaffolds were synthesized through a series of hybridization steps. Hybridization steps 1–6 were completed for detection of the red signal, which detects the C2 probe (BTV-10). To create the green signal, which detects the C1 probe (BTV-17), hybridization steps 7–10 were performed (Supplementary Table S4). Steps 1–6 entailed washing each slide in 1X Wash Buffer, followed by the addition of 4 drops of the corresponding AMP reagent to each slide. Slides were incubated at 40 °C (30 min for AMP1, 15 min for AMP2, 30 min for AMP3, and 15 min for AMP4) or incubated at room temperature (30 min for AMP5 and 15 min for AMP6). Detection of the red signal, which detects the C2 probe (BTV-10), was accomplished by application of a 1:60 ratio of Red-B and Red-A reagents to each slide for an incubation period of 10 min at room temperature. Afterward, the slides were washed twice in 1X Wash Buffer in preparation for hybridization steps 7–10 for green signal detection.

For hybridization steps 7–10, 4 drops of the corresponding AMP reagent were applied to each slide and then incubated at 40 °C (15 min for AMP7 and 30 min for AMP8) or incubated at room temperature (30 min for AMP9 and 15 min AMP10). After each hybridization step, the slides were washed twice in 1X Wash Buffer. Detection of the green signal, which detects the C1 probe (BTV-17), was accomplished by application of 1:50 ratio of Green-B and Green-A reagents to each slide for an incubation period of 10 min at room temperature followed by two washes in 1X Wash Buffer. Slides were counterstained in 50% Gill’s hematoxylin for 30 s. To remove excess stain, slides were immersed in tap water several times and incubated for 15 min at 60 °C to dry. After 5 min of cooling at room temperature, slides were briefly dipped into xylene abd then mounted using VectaMount Mounting Medium (Vector Laboratories, Inc., Burlingame, CA, USA, REF H-5000), after which a coverslip was placed over the section.

### 2.4. Image Capture

Images of histological slides were visualized on an Olympus BX53 microscope and captured with an attached Olympus DP28 camera. Images were processed using Olympus cellSens Entry software.

## 3. Results

### 3.1. Identification of C. sonorensis Structures in Hematoxylin-and-Eosin-Stained Slides

**Figure 1:**
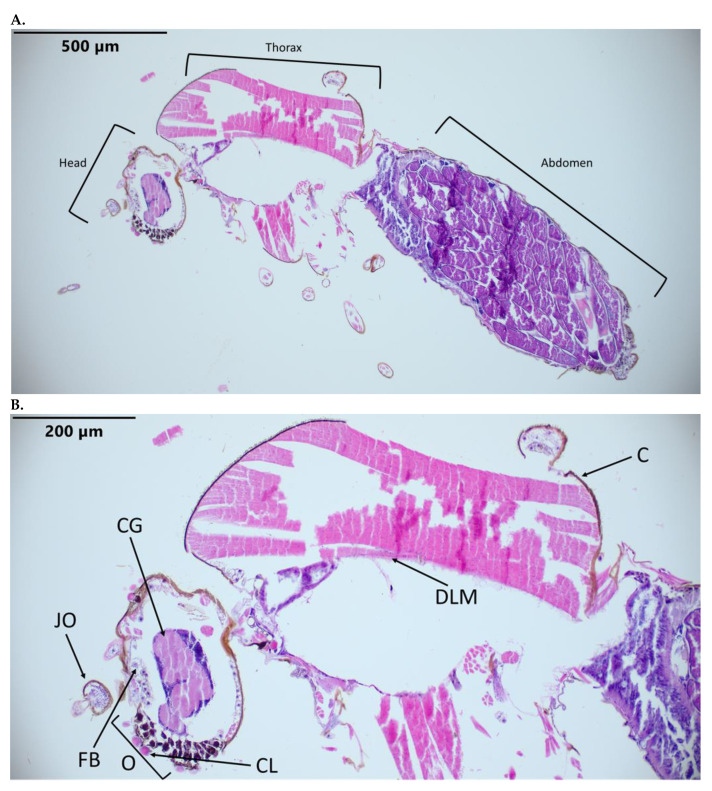
*C. sonorensis* sections stained with hematoxylin and eosin. (**A**) *C. sonorensis* at 10×. (**B**) Head and thorax at 20×. Labeled tissues: JO, Johnston’s organ; O, ommatidia; CL, corneal lens; FB, fat body; C, cuticle; CG, cerebral ganglion; DLM, dorsal longitudinal muscle.

Hematoxylin-and-eosin-stained slides of *C. sonorensis* facilitated the identification of anatomical structures as a reference to evaluate BTV virus dissemination (Figure 1). Routinely observed structures include the dorsal longitudinal muscles, midgut, ovarian follicles, overlaying follicular epithelium and cuticle. Additionally, the Johnston’s organ, cerebral ganglion, ommatidia, salivary glands, fat body, and tracheal cuticle are evident in some cross sections.

### 3.2. Control Probes Indicate Successful RNAscope Hybridization and Probe Detection

**Figure 2:**
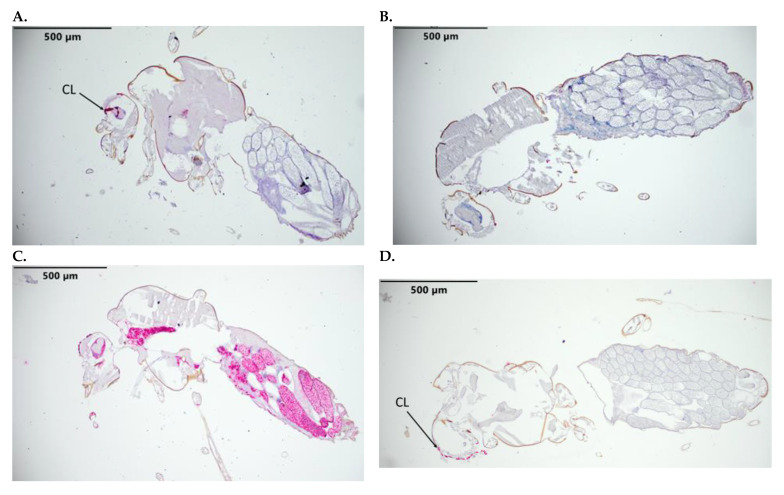
*C. sonorensis* sections stained with RNAscope control probes. (**A**) Negative control probe (bacterial DapB RNA). (**B**) Positive Channel 1 control (Cso-TSA-m4178) stained with green. (**C**) Positive Channel 2 control probe (Cso-V-ATPase16) stained with Fast Red. (**D**) BTV-10 (C2) and BTV-17 (C1) segment 2 specific probes (mock-infected). Labeled tissue: CL, corneal lens with nonspecific staining.

Successful RNAscope hybridization and probe detection were demonstrated by the application of control probes. The negative control probe designed for the DapB gene exhibited no staining, with the exception of some non-specific staining of the ommatidia. The C1 and C2 positive control host probes (for the detection of EF1b and vacuoloar ATPase housekeeping genes, respectively) exhibited green and red staining, respectively (Figure 2). None of the mock-infected *C. sonorensis* exhibited staining for BTV-17 or BTV-10, with the exception of the non-specific red staining of the corneal lens of the ommatidia.

### 3.3. Detection and Distribution of BTV-10 and BTV-17 in Single Infection Treatment Groups

**Figure 3:**
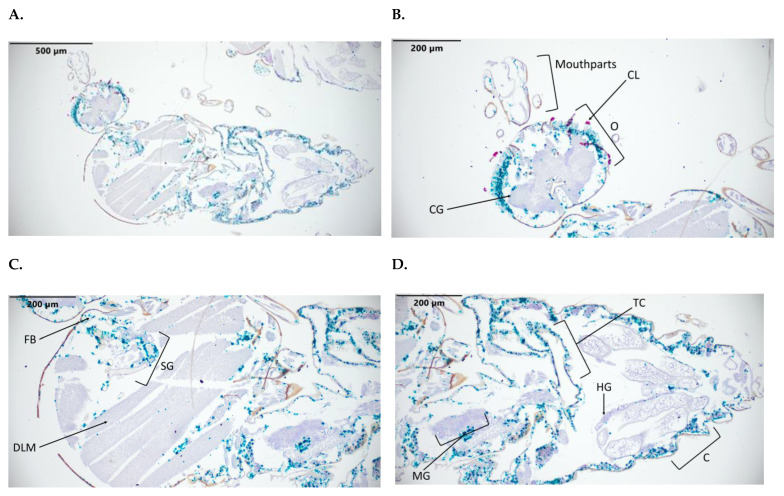

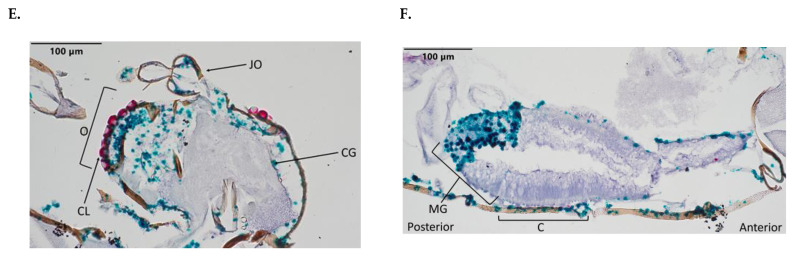
*C. sonorensis* 10 days post BTV-17 blood meal sections. Stained with BTV-10 and BTV-17 segment 2 probes. BTV-10 probe (Channel 2) binding is red staining, and BTV-17 probe (Channel 1) binding is green staining. (**A**) Full body at 10×. (**B**) Head at 20×. (**C**) Thorax at 20×. (**D**) Abdomen at 20×. (**E**) Head at 40×. (**F**) Abdomen at 40×. Labeled tissues: JO, Johnston’s organ; O, ommatidia; CL, corneal lens; CG, cerebral ganglion; FB, fat body; SG, salivary gland; DLM, dorsal longitudinal muscle; C, cuticle; TC, tracheal cuticle; MG, midgut; HG, hindgut.

*C. sonorensis* fed a BTV-17-spiked blood meal had a staining detection of 8 out of 13 midges (Table 1). All specimens with detection of BTV-17 had virus dissemination beyond the midgut. Moderate-to-strong staining of BTV-17 was notable in the basal areas of the ommatidia, periphery of the cerebral ganglion, salivary gland, tracheal cuticle, midgut, follicular epithelium, fat body, and cuticle. There was no detection of BTV-17 in the ovarian follicles. There was non-specific red staining of the cornea of the ommatidia (Figure 3 and Table 2).

**Figure 4:**
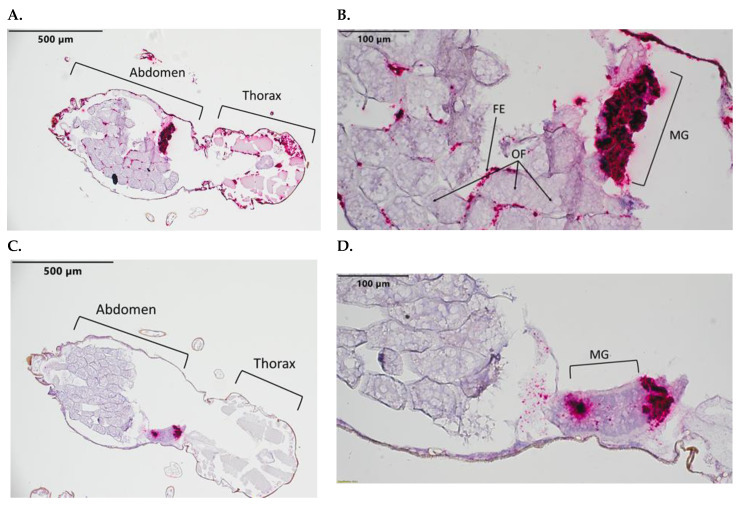
*C. sonorensis* 10 days post BTV-10 blood meal sections. Stained with BTV-10 and BTV-17 segment 2 probes. BTV-10 probe (Channel 2) binding is red staining. (**A**) Full body at 10×. (**B**) Abdomen at 40×. (**C**) Full body at 10× with limited BTV dissemination. (**D**) Abdomen at 40× of same specimen from (**C**). Labeled tissues: MG, midgut; OF, ovarian follicles; FE, follicular epithelium.

*C. sonorensis* fed a BTV-10-spiked blood meal had a staining detection of 2 out of 15 midges (Table 1). There was evidence of the dissemination of BTV-10 beyond the midgut in one specimen, with the presence of BTV-10 evident in the salivary gland, follicular epithelium, fat body, and cuticle. BTV-10 was absent in the ovarian follicles (Figure 4). However, the second specimen with BTV-10 detection had most of the virus localized to the midgut, with intensive staining (Figure 4C,D and Table 2).

### 3.4. Detection and Distribution of BTV-10 and BTV-17 in Co-Infection Treatment Group

**Figure 5:**
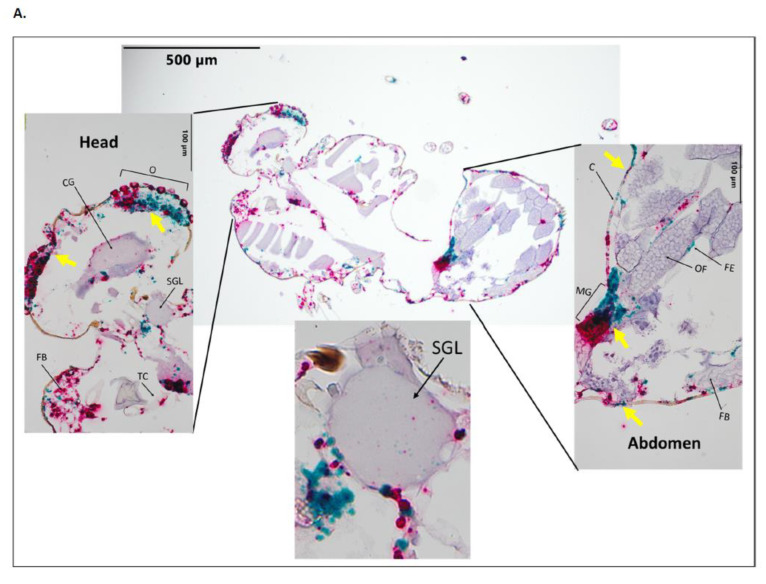

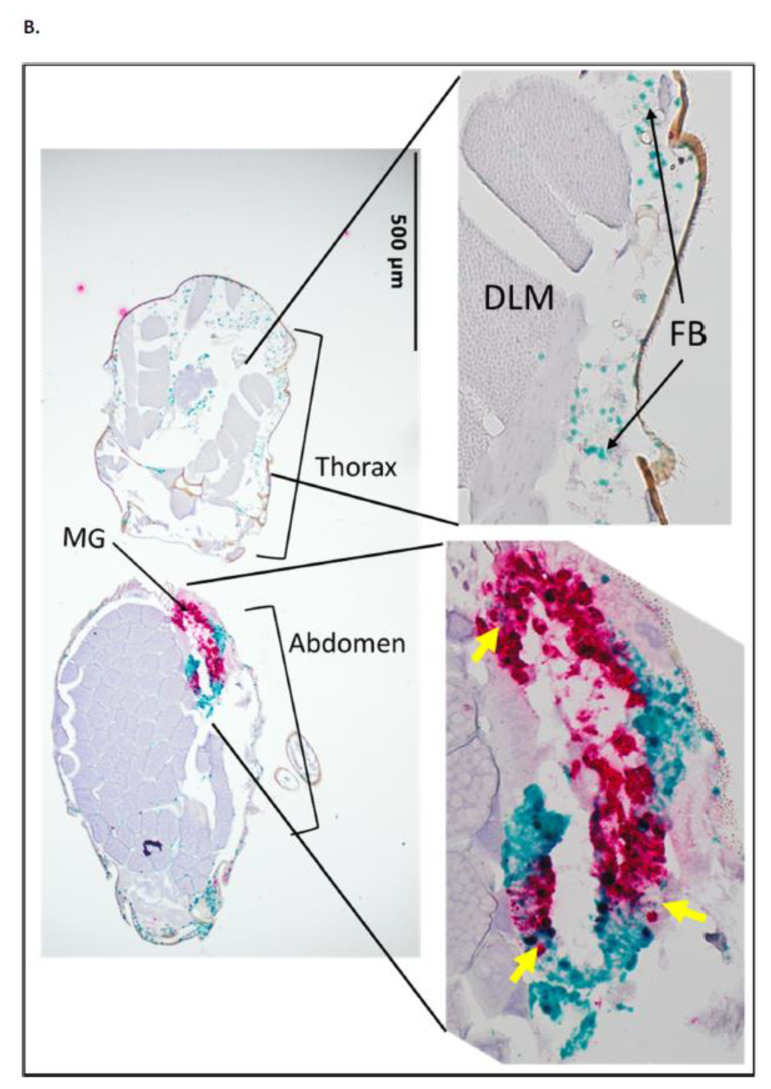

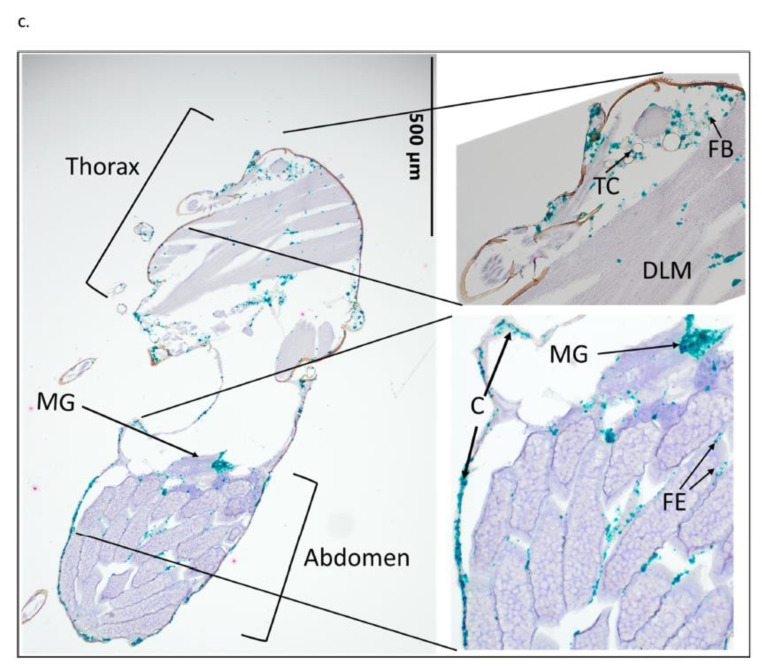
Sections from *C. sonorensis* collected 10 days after combined BTV-10 and BTV-17 blood meal. Stained with BTV-10 and BTV-17 segment 2 specific probes. BTV-10 probe (Channel 2) binding is red staining, and BTV-17 probe (Channel 1) binding is green staining. Yellow arrows indicate examples of overlap of red and green staining. (**A**) Disseminated BTV-10 and BTV-17 co-infection at 10× with head, salivary gland lumen, and abdomen at 40×. (**B**) Disseminated BTV-17 with BTV-10 localizing to midgut at 10× and thorax and abdomen at 40× (**C**) Disseminated BTV-17 with BTV-10 absent at 10× and thorax and abdomen at 40× Labeled tissues: O, ommatidia; CG, cerebral ganglion; FB, fat body; TC, tracheal cuticle; SGL, salivary gland lumen; C, cuticle; CL, corneal lens; DLM, dorsal longitudinal muscle; MG, midgut; OF, ovarian follicle; FE, follicular epithelium.

Both BTV-17 and BTV-10 were present in 3 of 14 midges, while 3 of 14 midges only exhibited BTV-17, and 1 of 14 midges only exhibited BTV-10 (Table 1). Individual *C. sonorenesis* demonstrated varied patterns of BTV-10 and BTV-17 distribution. Two of the midges with both BTV-10 and BTV-17 detection had evidence of BTV-10 and BTV-17 dissemination (Figure 5A and Table 3). In contrast, Figure 5B demonstrates a co-infected midge with dissemination of BTV-17 and with BTV-10 mostly confined to the midgut (unfortunately, the head was not captured in the cross section, demonstrating the challenges in performing histology on such small specimens). Figure 5C is an example where only BTV-17 was detected. Interestingly, while BTV-17 and BTV-10 demonstrated a similar tissue tropism, they often presented in a mosaic pattern with limited overlapping (Figure 5). Tissues where both BTV serotypes were detected included the ommatidia, cerebral ganglion, salivary gland (including a small presence of both BTV serotypes in the salivary lumen), tracheal cuticle, midgut, follicular epithelium, fat body, and cuticle. The visible overlap of both serotypes in tissues was most prominent in parts of the ommatidia, midgut, and cuticle. For both serotypes, BTV was apparent in the follicular epithelium but absent in the ovarian follicles.

## 4. Discussion

BTV co-infection in the *C. sonorensis* vector is substantiated by the application of a chromogenic duplex ISH (RNAscope) platform. Moreover, ISH is a promising approach for the detection of BTV in formalin-fixed and paraffin-embedded (FFPE) *C. sonorensis* specimens. While a convenient and abundant archived sample type, FFPE tissue is regarded as a challenging sample type for the detection of BTV via IHC approaches [36,37,38]. IHC relies on the known characterization, which is limited, of BTV antibodies to bind to BTV proteins for detection. Additionally, formaldehyde treatment can create cross-linking of epitopes. ISH detection utilizes the specific target genome sequence for the design of its probes. With the advancement of next-generation sequencing platforms, the database of BTV sequences is expanding.

In *C. sonorensis* exposed to both BTV-10 and BTV-17, it is important to interpret probe detection in the context of potential reassortment, as the probes only detect the nucleic acid of segment 2 from each serotype. Thus, while segment 2 is detected, the corresponding virion could potentially have nucleic segment contributions for the remaining nine segments from either parental strain. As BTV has the potential to drastically evolve via reassortment, understanding of the viral dissemination and cellular co-infection in the *C. sonorensis* vector, which represent current knowledge gaps, could be input to inform predictive modeling and enhance the understanding of reassortment mechanisms and barriers [21]. ISH facilitates the visualization of the dissemination and extent of serotype overlap in midges, which could assist in refining the reassortment model.

Important barriers to BTV dissemination in *C. sonorensis* were identified, including the mesenteron infection barrier (prevents BTV infection of the midgut), mesenteron escape barrier (limits BTV to the gut cells), and dissemination barrier (prevents BTV in the haemocoel from infecting other tissues) [39]. In one single infected and one co-infected midge, BTV-10 detection was limited to the midgut without dissemination (Figure 4C,D and Figure 5B). When BTV-17 segment 2 was detected, it always exhibited dissemination beyond the midgut in *C. sonorensis*. Perhaps BTV-17 (or corresponding strains/reassortants) confers an advantage within the midge host, while BTV-10 has difficulties traversing the mesenteron escape barrier.

The detection of BTV in the salivary gland is notable as this is the route by which BTV is transmitted to the ruminant host (Figure 3C and Figure 5A). Currently, a salivary gland infection barrier and a salivary gland escape barrier for BTV are not recognized in *C. sonorensis* [39]. In these cross sections, a small amount of virus was detected in the lumen of the salivary gland. An interesting possibility is whether the act of feeding stimulates the release of virus into the salivary gland lumen, which is then transmitted to the mammalian host via the saliva. Previous studies on the ultrastructure of *Musca domestica* salivary glands infected with *Musca domestica* salivary gland hypertrophy virus (MdSGHV) indicated that there is a cuticular, chitinous lining between the secretory cells and salivary duct lumen that may require disruption by MdSGHV for the virus to access the lumen. Further study of the dissemination mechanisms of BTV into the salivary gland lumen in a variety of BTV competent *Culicoides* spp. would be informative for understanding BTV transmission [40].

When both BTV-10 and BTV-17 segment 2 were present, there was mostly a mosaic staining pattern, suggesting that cellular co-infection between serotypes 10 and 17 at 10 days post-infection is not extensive. However, it is important to interpret this observation in the context of potential reassortment. While the BTV segment 2 target sequences may not exhibit extensive overlap in cells, the virus infecting cells could be reassortants between serotypes. This could be due to the occurrence of super infection exclusion, where once an infection is established in a cell it limits entry of a second virus. It was demonstrated that BTV can spread cell to cell as free virus particles or via extracellular vesicles containing numerous virus particles [41]. While extracellular vesicles are more efficient at establishing infection, the free virus particles are more able to overcome superinfection exclusion [41]. If the majority of BTV in the infectious blood meal was in the extracellular vesicle form, this could result in reduced initial cellular co-infection in *C. sonorenesis*. Furthermore, if the BTV egress from *C. sonorenesis* cells is mostly as extracellular vesicles, this could result in reduced cellular co-infection and explain the mosaic patterns between serotypes observed in co-infected *C. sonorenesis.* However, a fluorescence ISH platform could be employed for a better resolution when evaluating cellular co-infection.

Both virus serotypes had similar tissue tropisms and were routinely detected in the ommatidia, cerebral ganglion, fat body, tracheal cuticle, and cuticle. While pathological changes and metabolic alterations are difficult to assess in invertebrates via histology, the presence of virus in these tissues may provide a connection to understanding changes in an infected insect. Zika virus infection in the *Aedes aegypti* vector demonstrated neurotropism connected to behavioral changes that could be considered advantageous for virus transmission [42]. Notable changes in virus-infected insects include alterations in the midgut lipid metabolism in *Aedes aegypti* infected with dengue virus, deviations in the foraging behavior of *Solenopsis invicta* infected with Solenopsis invicta virus 3, and altered susceptibility of *Drosophila melanogaster* to certain bacterial and fungal pathogens when infected with Galbut virus [43,44,45]. Ichnovirus from *Hyposoter didymator* induces apoptosis in the fat body cells of *Spodoptera frugiperda* larvae, resulting in modulated immunity [46]. As more life trait and metabolomic studies elucidate the changes occurring in BTV-infected *C. sonorensis*, knowledge of BTV tissue tropism will assist in understanding the pathogenesis.

Similar to previous insect ISH studies, *C. sonorensis* specimens demonstrated non-specific red staining of the ommatidia [25]. Thus, caution must be exercised when interpreting viral tropism for eyes when using this stain. However, the abundant staining in infected midges compared to the negative controls and the presence of the green signal for BTV-17 in exposed midges indicate that there is viral tropism for the ommatidia. Prior studies using immunohistochemistry also confirmed a BTV and closely related epizootic hemorrhagic disease virus tropism for *C. sonorensis* ommatidia [47,48]. Viral tropism for insect ommatidia is evident in other arthropod-borne viruses including Rift Valley fever virus [25]. As BTV was detected in the ommatidia of the midge, there could possibly be an effect on midge behavior in response to visual cues, like light, which has implications for trapping and insect surveillance techniques. In addition to detecting BTV in the ommatidia, it has been suggested that midges with BTV may be UV-light-averse, compared to uninfected midges. A higher proportion of BTV-infected midges was found in CO_2_-only CDC suction traps, compared to CO2 traps equipped with lights [47]. In another study, the altered transcription of genes associated with sensory perception was demonstrated in *C. sonorensis* experimentally infected with epizootic hemorrhagic disease virus [49]. Thus, a virus in the cerebral ganglia could play a part in altering midge behavior.

The absence of viral detection in the midge ovarian follicles among all infection groups is consistent with the current understanding that BTV is not vertically transmitted in the invertebrate host [50]. However, there seems to be abundant amount of virus detection in the epithelium overlaying the ovarian follicles. While we were curious to investigate the presence or absence of virus detection in gravid midges to further validate vertical transmission studies, more adequate visualization of other abdominal structures (including more complete midgut and Malpighian tubules) may be accomplished with *C. sonorensis* that remain nulliparous.

*Culicoides* are extremely small-bodied insects, with average body lengths of ~1.5 mm, compared to other vector groups, like mosquitoes (e.g., *Culex pipiens* 4–10 mm), which made it a challenge to consistently obtain full cross sections [25,35,51]. However, an advantage to the ISH technique is that it facilitates viral detection in different tissues, as the dissection and isolation of distinct tissue types can be very limiting and difficult in an insect of this size. Another challenge was identifying the appropriate *C. sonorensis* housekeeping gene sequences for the positive control probes. Initial attempts at designing a positive control probe using the COX1 housekeeping gene was deemed not feasible by RNAscope engineers due to its short nucleotide sequence. Our positive probe design targets were, thus, informed by work that indicates that the selected targets are reliably expressed [33]. GAPDH is another potential target for a positive control design that is often used in mosquito work; however, a sequence specific to *Culicoides* species could not be identified in GenBank at the time this project was designed [25].

For the first time, an in situ hybridization (RNAscope) platform was successfully employed in *C. sonorensis* co-infected with two different BTV serotypes via spiked blood meal. This approach has the potential to facilitate the interrogation of BTV tissue tropism, dissemination, and cellular co-infection dynamics in the BTV insect vector and contribute to additional studies to further understand the ramifications of the reassortment and evolution of this virus of agricultural importance.

## Figures and Tables

**Table 1:** Percentage of *C. sonorensis* with detectable BTV-10 and BTV-17 segment 2. Numerator indicates number of midges with BTV detection, and denominator indicates number of midges processed for staining.

Infection Treatment Group	Detection of BTV-10 Segment 2 Only	Detection Of BTV-17 Segment 2 Only	Detection of BTV-10 and BTV-17 Segment 2	No Detection
BTV-10	2/15 (13.3%)	0/15 (0%)	0/15 (0%)	13/15 (86.7%)
BTV-17	0/13 (0%)	8/13 (61.5%)	0/13 (0%)	5/13 (38.5%)
BTV-10 and BTV-17	1/14 (7.1%)	3/14 (21.4%)	3/14 (21.4%)	7/14 (50.0%)

**Table 2:** BTV detection in single infection treatment groups. Numerator indicates number of midges with detection in tissue, and denominator indicates number of slides where tissue was present. n/a indicates that the tissue was not present. Intensity of staining is indicated by very weak (+), weak (++), moderate (+++), and strong (++++).

	BTV-10 Infection Treatment Group		BTV-17 Infection Treatment Group
	Detection of BTV-10		Detection of BTV-17
Number of Midges with BTV Detection	2 Out of 15 Midges		8 Out of 13 Midges
Dissemination Pattern	1 Midge with BTV-10 Dissemination	1 Midge with BTV-10 Localized to Midgut	8 Midges with BTV-17 Dissemination
**Tissue**			
Johnston’s organ	n/a	n/a	3/3 (++) to (+++)
ommatidia	n/a	n/a	7/7 (+++) to (++++)
cerebral ganglion	n/a	n/a	7/7 (++) to (+++) (periphery)
salivary gland	1/1 (+)	0/1	5/6 (++) to (+++)
dorsal longitudinal muscle	1/1 (+)	0/1	5/8 (+)
tracheal cuticle	n/a	n/a	8/8 (++) to (+++)
midgut	1/1 (++++)	1/1 (++++)	6/6 (+++) to (++++)
ovarian follicles	0/1	0/1	0/8
follicular epithelium	1/1 (+++)	0/1	6/8 (++) to (+++)
fat body	1/1 (++++)	0/1	8/8 (+++)
cuticle	1/1 (+++)	0/1	7/8 (++) to (++++)

**Table 3:** BTV detection in co-infection treatment group. Numerator indicates number of midges with detection in tissue, and denominator indicates number of slides where tissue was present. n/a indicates that the tissue was not present. Intensity of staining is indicated by very weak (+), weak (++), moderate (+++), and strong (++++).

		BTV-10 and BTV-17 Infection Treatment Group		
	Detection of BTV-10	Detection of BTV-17	Detection of BTV-10 and BTV-17	
Number of Midges with BTV Detection	1 Out of 14 Midges	3 Out of 14 Midges	3 Out of 14 Midges	
Dissemination Pattern	1 Midge with BTV-10 Dissemination	3 Midges with BTV-17 Dissemination	2 Midges with BTV-10 and 17 Dissemination	1 Midge with BTV-17 Dissemination and BTV-10 Localized to Midgut
**Tissue/Organ**				
Johnston’s organ	n/a	1/1 (++)	n/a	n/a
ommatidia	n/a	2/2 (++) to (++++)	2/2 (BTV-10 and 17) (++) to (+++)	n/a
cerebral ganglion	n/a	2/2 (++) to (+++)	2/2 (BTV-10 and 17) (++) to (+++)	n/a
salivary gland	n/a	2/2 (+)	2/2 (BTV-10 and 17) (++)	n/a
dorsal longitudinal muscle	1/1 (+)	3/3 (+)	2/2 (BTV-10 and 17) (+)	1/1 (BTV-17 only) (+)
tracheal cuticle	n/a	3/3 (++) to (+++)	2/2 (BTV-10 and 17) (++)	1/1 (BTV-17 only) (++)
midgut	1/1 (+++)	2/2 (+++) to (++++)	1/2 (BTV-10 and 17) (+++) to (++++)	1/1 (BTV-10 and 17) (++++)
ovarian follicles	n/a	0/3	0/2	0/1
follicular epithelium	n/a	3/3 (++) to (+++)	2/2 (BTV-10 and 17) (++)	1/1 (BTV-17 only) (+)
fat body	1/1 (+++)	3/3 (++) to (++++)	2/2 (BTV-10 and 17) (+++)	1/1 (BTV-17 only) (+++)
cuticle	1/1 (+++)	3/3 (++) to (++++)	2/2 (BTV-10 and 17) (++) to (+++)	1/1 (BTV-17 only) (++)

## Data Availability

Data are contained within the article or supplementary material.

## Supplementary Materials

The following supporting information can be downloaded at https://www.mdpi.com/article/10.3390/pathogens12101207/s1, Table S1: BTV TCID_50_/mL in blood meal for infection groups; Table S2: GenBank accession # for BTV-17 & BTV-10 segments; Table S3: Probe targets; and Table S4: Hybridization and signal detection steps.
