# Supplementary material for: In Situ Hybridization (RNAscope) Detection of Bluetongue Virus Serotypes 10 and 17 in Experimentally Co-Infected Culicoides sonorensis
Source: Pathogens. 2023 Sep 30;12(10):1207. doi: 10.3390/pathogens12101207 (PMC10609982; doi:10.3390/pathogens12101207)
Supplement: Supplementary file 1 [file pathogens-12-01207-s001.zip › pathogens-2577884-supplementary.pdf]

Table S1. BTV $TCID_{50}$/mL in blood meal for infection groups.

Treatment Group	BTV-10 $TCID_{50}$/mL	BTV-17 $TCID_{50}$/mL	Total $TCID_{50}$/mL
**Negative control blood meal**	N/A	N/A	N/A
**BTV-10 blood meal**	1×10^5	N/A	1×10^5
**BTV-17 blood meal**	N/A	1×10^5	1×10^5
**BTV-10 & BTV-17 blood meal**	5×10^4	5×10^4	1×10^5

Table S2. GenBank accession # for BTV-17 & BTV-10 segments.

Nucleic Acid Segment	BTV-17 CO 2018	BTV-10 ATCC CA 1952
1	OQ798198	MW456747
2	OQ798199	MW456748
3	OQ798200	MW456749
4	OQ798201	MW456750
5	OQ798202	MW456751
6	OQ798203	MW456752
7	OQ798204	MW456753
8	OQ798205	MW456754
9	OQ798206	MW456755
10	OQ798207	MW456756

Table S3. Probe targets.

Probe	Probe Description	NCBI sequence	Nucleotide position
**Negative**	DapB	ACD provided	ACD provided
**Positive Control C1**	Cso-TSA-m41748	GAWM01010754	2-954
**Positive Control C2**	Cso-V-ATPase16	AY752855	2-668
**C1**	V-BTV17-segment2-VP2-C1	OQ798199	1801-2839
**C2**	V-BTV10-segment2-VP2-C2	JQ740772 and MW456748	184 – 1168

Table S4. Hybridization and signal detection steps

Hybridization reagent	Incubation Temp	Incubation Time
**AMP1**	40° C	30min
**AMP2**	40° C	15min
**AMP3**	40° C	30min
**AMP4**	40° C	15min
**AMP5**	Room Temp	30min
**AMP6**	Room Temp	15min
**C2 (red) Signal detection**	Room Temp	10min
**AMP7**	40° C	15min
**AMP8**	40° C	30min
**AMP9**	Room Temp	30min
**AMP10**	Room Temp	15min
**C1 (green) Signal detection**	Room Temp	10min
